# Effect of seasonal variation on yield and leaf quality of tea clone (Camellia sinensis (L.) O. Kuntze) in South West Ethiopia

**DOI:** 10.1016/j.heliyon.2023.e14051

**Published:** 2023-02-24

**Authors:** Tesfaye Benti, Adugna Debela, Yetenayet Bekele, Sultan Suleman

**Affiliations:** a Department of Horticulture College of Agriculture in Mizan Tepi University Southwest Ethiopia, Ethiopia; b Ethiopia Coffee and Tea Authority, Oromia, Ethiopia; c Department of Postharvest Management of Jimma University College of Agriculture, Oromia, Ethiopia; d Jimma University Laboratory of Drug Quality (JuLaDQ) and School of Pharmacy, Jimma University, Oromia, Ethiopia

**Keywords:** Climate, Rainfall, Season, Shoot replacement, Temperature

## Abstract

The tea plant is widely cultivated in southwest Ethiopia. But the impact of seasonal variation on monthly yield, leaf quality, and the long-term yield response potential of clones has not been studied. The objective of the study is to determine the impact of seasonal change and climate variables on the yield and leaf quality of tea plants in southwest Ethiopia. The experiment consisted of five clones and four seasons under a split-plot design and was replicated three times. The results indicated that the yield and leaf quality showed significant variation in the different seasons at P < 0.05. The highest peak yields of 12.68, 12.59, and 11.3 kg plot^−1^ were recorded in May, June, and April, respectively, and the yield suddenly dropped by 5.1% in July. Then the soft banjhi increased by 5–10% in July. The yield response potential of clones is highly affected by monthly climate variation at P < 0.05. Clone BB-35 recorded the highest (18.8 kg plot^−1^) yield in June, followed by clones 11/4 (18.3) in May, 11/56 (14.7) in November, 6/8 (11.7) in December, and 12/38 (5.78 kg plot^−1^) in June. The lowest mean green leaf and a longer shoot replacement cycle were created due to a decrease in rainfall to 760 mm/month and rising temperatures above 26.35 °C in winter. The leaf phenological response of tea clones is strongly governed by the monthly temperature and suitable precipitation pattern of a season. The highlands have two harvesting seasons, i.e., a dry and a wet harvesting season. The dry harvesting season, which exists between the middle of December and March, accounts for 18.3–24.3% of the total annual yield. The wet harvesting season is subdivided further into two peak harvesting seasons. The first harvest is characterized by a short plucking round, and the highest peak yield occurs in April, May, and June, accounting for 40.22–42.2% of the total annual yield. The second wet harvesting season begins in September and ends in the middle of December, contributing to 35.5–40% of the annual yield. Seasonal variation has a direct impact on leaf quality and clone yielding potential. Clones show higher yield and shorter plucking rounds at maximum temperatures above 23.03 °C and below 26.35 °C, but temperatures above 28.34 °C and below 10.38 °C have a negative effect on leaf quality and yield. Over the last two decades, rainfall, maximum, and mean temperatures all increased by 16.09 mm y-1, 0.127 °C, and 0.0566 °C y^−1^, respectively, and the tea plant showed a strong correlation with maximum temperature (76%), whereas mean temperature (44.6%) and annual rainfall (32.8%) correlated weakly. Green leaf production is well explained by around 85.4% of the observed climate variance, with an increase of 1287.18 tonnes y^−1^, and highland tea production will exhibit a positive net benefit from expected climate change in the future.

## 1 Introduction

Tea grows in a wide range of latitudes between 45° N and 34° S, which covers around 58 countries [1]. The cultivation of tea is restricted to tropical and subtropical climates [2, 3], because warm temperatures and adequate rainfall are critical climatic factors for successful tea cultivation [4, 5]. The world tea production increased by 3.5% annually over the last decade to reach 6.29 million tonnes in 2020 [6]. China produced approximately 3.12 million tons and exported 2.30 billion U.S. dollars' worth of tea in 2021 [7].

Ethiopia's tea plantations are primarily concentrated in the southwest highlands. The area is located in the tropical zone lying between the Equator and the Tropic of Cancer, with a diversified agro-climate that is more suitable for cultivating perennial milled-stimulating plants like coffee, tea, and khat (Catha edulis), and Camellia sinensis var. assamica adapted and thrived well in the area [8]. Climate change is a major factor influencing the productivity of perennial cash crops in the region because tea and coffee are more sensitive to climate change [9, 10, 11]^.^

Climate change triggered by global warming poses a major threat to agriculture systems [12]. Temperatures are expected to rise by more than 1.5 °C over the next 20 years [13]. East Africa's temperatures are expected to rise by about 2.5 °C by 2025 and 3.4 °C by 2075, while rainfall is expected to rise by about 2% by 2025 and 11% by 2075 [14]. Such a rise in global warming will cause a threat to the production of sensitive cash crops like coffee and tea; hence, the existing suitable growing areas in Uganda will be reduced by 20–40 % [15], 35–55% in Kenya [16], 39–59% in Ethiopia [11, 17], and 8–17% in Sri Lanka [18]. In general, East African countries will reduce yields by 40–50% due to this rise in temperatures [17, 19]. Also, the production of major cash crops shows an increment at high latitudes while their suitability decreases at low latitudes [20, 21]. Climate change creates losses or gains, shifting the timing of seasons, disrupting tea productivity, and affecting the quality of tea production [22, 23, 24, 25, 26, 27].

In fact, major coffee and tea-producing countries have experienced rising temperatures; average maximum temperatures and rainfall distribution patterns have shifted dramatically in recent years [28, 29, 30, 31]^.^ Ethiopia's average temperature has increased by 1–1.3 °C since 1960, at an average rate of 0.25–0.28 °C per decade [32, 33], and will have risen by 1.7–2.1 °C by 2050 [34]. Global warming is expected to have a significant impact on tea production, which is most likely to have an impact on both the quantity and quality of tea [10, 28]. Ethiopian green tea contains high levels of polyphenols (24.17–30.82 mg GAE/g) and a small amount of caffeine (1.82–3.06%) [8]. Total polyphenols in Chinese black tea, green tea, and oolong tea ranged from 13.6% to 23.7%, 11.6%–27.5%, and 14.9%–24.5%, respectively [35]. The N, P, and K content of tea leaves is affected by seasonal variation. The spring tea leaves have greater nutrient content than summer and autumn tea leaves [36].

The first commercial tea plantation was established on 25 ha in 1957; by 2020, it had expanded to 3175.31 ha and was producing 7767.6 tonnes y^−1^ on average [8]. The southwest highlands experience uniform rainfall distribution [37], and the annual and seasonal precipitations are decreasing while maximum and minimum temperatures are rising by 0.4 and 0.3 °C per decade, respectively [38]. However, for the past 60 years, the impact of seasonal change and variation on the yield and leaf quality responses of tea clones and their long-term seasonal response patterns have not been studied. Due to this, the tea industry faces challenges in determining the specific timing of the main agronomical activities, i.e., preparation and application of inputs; hiring temporary workers for peak harvesting season; and identifying the exact annual pruning, monthly bush care, and specific growth pattern of clones with current climate variables. In order to achieve the objective of commercial cultivation, long-year climate variables and the seasonal yield and leaf quality response patterns of the clones are examined based on monthly climate variation in the growth area. Understanding the actual timing of phenological events is critical for agriculture and other fields [39]; hence, phenological timing is necessary to schedule major agronomic activities and model bioclimatic events for adaptive management.

In order to develop a yield response model for the tea plant in the growth area, the long-term climate variables were examined and compared with the long-term, monthly yields, and leaf quality of tea plants. The long-term bioclimatic modeling of species based on climate change is suitable to assess the effect of seasonal change on species and can be used to predict and make decisions for expected climate change in the near future [40, 41]. This study provides a better understanding of the impact of recent and past seasonal fluctuations and climate variability on the yield and leaf quality responses of tea plants. Additionally, the development of mitigation measures to ensure the plant's long-term viability as well as long-term investments with its key players in the sector are also greatly aided by improvement of various agronomical practices based on the growth model of the tea plant.

## 2 Material and method

### 2.1 The study area and size

**Figure 1:**
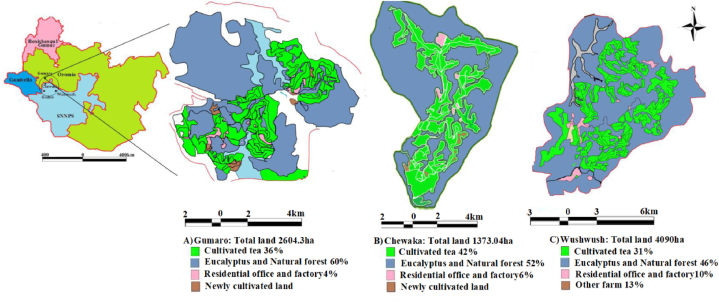
Map of Ethiopia tea plantation.

The study was carried out in southwest Ethiopia between the latitudes of 7° 18′N and 8° 08′N and the longitudes of 36° E and 35° 27′E, at the meeting point of the Oromia, Gambella, and southwest regions of Ethiopia (Fig. 1), with different altitude ranges from 1700 to 2350 m above sea level. The study area received annual rainfall of 1640–2000 mm and has eight long wet months (March–November) and four dry months (December-mid-March), with an average temperature of 25 °C (max.) and 12 °C (min.) [8, 42]. The study area has a cool variant of the tropical climate with four seasons [37]**,** including autumn, locally known as "Belg," which has three wet seasons (September, October, and November). Next is winter "Bega," which is a dry season that includes December, January, and February. Then came spring, "Tsedey," the hottest season (March, April, and May), and the final season is summer, "Kiremt," which includes June, July, and August [8, 38, 42, 43].

### 2.2 Experimental design

The experiment was carried out in a split-plot arrangement with clones as the main plot and seasons as sub-plot factors; the main plot has five clones (6/8, BB-35, 11/56, 11/4, and 12/38), and the subplot has four seasons (autumn, winter, spring, and summer) and is replicated three times. The 28-year-old tea bushes were chosen and pruned one year before the experiment, and the plot has four rows with a total size of 6.0 m × 3.6 m, spaced at 1.2 m × 0.6 m on an established tea farm. Then the perennial weeds are removed, and all the phosphate and potassium are applied once at the beginning of the rainy season at a rate of 150 kg ha^−1^ as NPK 20:10:10. The first split nitrogen application begins in April before the soil moisture reaches its saturation point; the remaining nitrogen is applied between July and August according to a factory's recommendation. All available leaves were harvested by hand at seven-day intervals to estimate the total plot yield (kg plot^−1^); the following data (shoot quality percentage, shoot replacement cycle, and number of plucking rounds) were recorded between 2019 and 2020. And the long-term yield, rainfall, and temperature data were collected from 1997 to 2018 from each tea farm.

### 2.3 Method of data collection

#### 2.3.1 Monthly yield

During the experiment years, the whole green leaves (kg plot ^−1^) were taken to evaluate the monthly yield potential of each clone. The tea yield is determined by the area harvested and the weight of the tea leaves plucked [26].

#### 2.3.2 Seasonal green leaf quality

Randomly, 100 g of a sample was taken from each plot to determine the composition of the shoot: as a bud +1, a bud +2, a bud +3, a bud +4 leaf, Soft and hard bhanji, half-cut leaves, quarter-cut leaves, broken pieces of stems, and the acceptable leaves are separated from the bad ones according to the standards of the factory, and the weight of a good tea leaf is expressed as a quality percentage of the sample, and the remaining shoot components are weighed, and their mass is expressed as a percentage of the poor tea leaves [44]. The best quality of green leaves must be greater than 70% [45].

#### 2.3.3 Shoot replacement cycle

Twenty active buds from each clone tag at each time after plucking, and the number of days is counted until the axillary bud grows into a shoot and the unfurling of true leaves reaches the third leaf [46].

#### 2.3.4 Long-year climate variables and yield

In the last twenty years (1997–2018), the meteorological data and yields were collected from each tea farm as follows:

*Yield at the national level*: Each year, descriptive statistics (minimum, maximum, and mean) of fresh leaf and made tea are computed at the national level, and the mean is calculated as the sum of the annual yields [Y_f_ (t)] at farm f, divided by the number of years.

*Yield at tea farm level*: Each year, descriptive statistics of fresh and made tea are calculated at the tea farm level, and the mean is calculated as the sum of the annual yields [Y_f_ (s, t)] for each tea farm f, divided by the number of years.

*Temperature:* Each year, temperature data is gathered at the tea farm level. The monthly mean temperature was related to the sum of the monthly yields at the tea farm level.

*Rainfall:* A linear regression analysis was used to relate the annual yield to the annual rainfall data obtained at the tea farm level (Y_f_):

Y_f_ (t) = β_0_ + β_1_ ∙ rain (t) + ε(t)

where Y_f_ (t) is the tea farm's specific yield for year (t) = 1997–2018, rain r (t) is the rainfall in region r in which the tea farm is located, and ε(t) is the error.

## 3 Result and discussion

### 3.1 Monthly yields patter of clones

**Figure 2:**
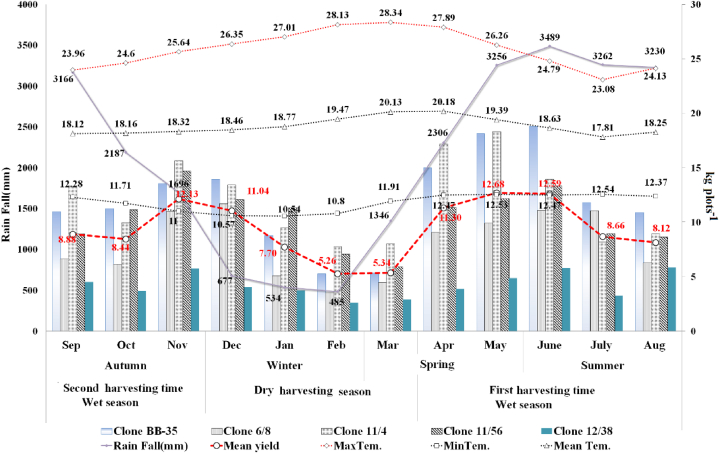
Average climate and seasonal yield response pattern of clone in southwest Ethiopia.

The average monthly yield response pattern of clones is presented in Fig. 2, and the yield response potential of clones is highly affected by monthly climate variation at P < 0.05. Clone BB-35 recorded the highest mean green leaf (18.8 kg plot^−1^) in June, followed by clones 11/4 (18.3) in May, 11/56 (14.7) in November, 6/8 (11.7) in December, and 12/38 (5.78) kg plot^−1^ in June. The lowest (2.6, 3.56, 5.27, 7.08, and 7.77 kg plot^−1^) was recorded by clones 12/38, 6/8, BB-35, 11/56, and 11/4 in February, respectively.

The first wet harvesting season began in the middle of March, when the annual mean rainfall increased by 942.1 mm/month (3078.25 mm) and the maximum temperature decreased slightly from 28.34 to 23.08 °C. Clones recorded the highest peak yields of 12.68, 12.59, and 11.3 kg plot^−1^ in May, June, and April, respectively. Then, the average maximum temperature dropped to 23.08 °C, clones entered a short resting phase in July, and yields rapidly dropped by 3.93 kg (8.67 and 8.12 kg plot^−1^) in July and August, respectively (Fig. 2). In July, large amounts of precipitation have a negative effect on tea yields [10].

The second wet harvesting season starts in early September and ends in the middle of December, with a gradual decrease in mean rainfall to 677 mm and an increase in maximum temperatures to 26.35 °C in December. The highest peak yields of 12.12 and 11.04 kg plot^−1^ were recorded in November and December, followed by 8.88 and 8.44 kg plot^−1^ in September and October, and the amount and duration of early-out rainfall that occurred in November and December had a significant impact on the yields.

**Figure 3:**
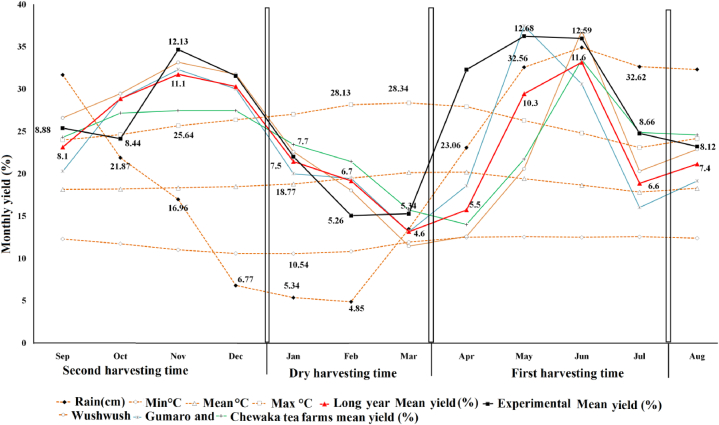
Long-term monthly yields response pattern of tea plant in southwest Ethiopia.

The third dry harvesting season existed between the middle of December and the middle of March. The rainfall decreased to 760.5 mm with a rise in average maximum temperatures (27.01, 28.13, and 28.34 °C) in January, February, and March, respectively (Fig. 2). Clones show a high yield reduction associated with maximum temperatures, and the lowest mean yields (2.6, 3.56, 5.22, 7.08, and 7.77 kg plot^−1^) were recorded in February by the clones 12/38, 6/8, BB-35, 11/56, and 11/4. Clones exhibited similar yield decline trends with a drop in rainfall and the opposite trend with a rise in temperature above 26.35 °C (Fig. 2). Similarly, monthly temperatures above 26.6 °C had a negative impact on tea yield [10], and the pattern, distribution, and frequency of monsoonal rainfall greatly affect the quality of tea as well as the quantity of yield [29]. The yield potential of clones is directly related to the monthly temperature and rainfall pattern of the growth area (Figure 2, Figure 3). Climate changes, mainly rain, are affecting tea yields, and the production strongly depends on stable rainfall and temperature compared to other crops [47, 48]. An extra one degree of warming at an average monthly temperature of 28 °C would result in a 3.8% reduction in yield [10]. Every day of extreme heat (34 °C < T mean <36 °C) reduces yields by 3.7% [49].

### 3.2 Seasonal green leaf quality

Seasonal variations influenced the leaf quality percentage of clones significantly (P < 0.05), and the highest autumn leaf quality scores (74.13 and 72.04%) were in September and November, with a low 70% leaf quality score in October. Hence, decreases in nighttime temperatures, morning fog, and cold dew are commonly observed in October. These phenomena damage the internal organelles of leaves, which lead to fermentation and a reduction in the quality of the leaves. In the case of clones, the highest mean leaf quality scores of 76.5, 72.8, 70.6, 70.4, and 69.9% were scored by clones 11/4, BB-35, 6/8, 12/38, and 11/56 in autumn, respectively (Table 1). Leaf quality and yield are gradually declining as maximum temperatures rise and minimum temperatures decrease during the transition period between autumn and winter. Similarly, tea production decreases with the increase in maximum and minimum temperatures [50].

**Table 1:** The average good leaf quality percentage of clones (2019 and 2020).

Harvesting season			Clone BB-35	Clone 6/8	Clone 11/4	Clone 11/56	Clone 12/38	Mean	C.V. (%)	LSD (≤0.05)
2^nd^ peak harvest	Autumn	Sep	73.2^ab^	73.47^ab^	76.93^a^	72.13^b^	74.9^ab^	74.13	11.49	3.94
		Oct	70.2^b^	66.3^c^	76.93^a^	68.3^bc^	68.13^bc^	70.0	8.69	2.3
		Nov	74.9^a^	72.2^b^	75.7^a^	69.2^c^	68.1^c^	72.04	8.55	1.97
	Mean		72.8^b^	70.6^cb^	76.5^a^	69.9^c^	70.4^c^	72.0	8.75	2.21
Dry season	Winter	Dec	72.53^b^	70.1^cb^	76.4^a^	68.5^c^	69.7^cb^	71.45	8.21	3.24
		Jan	68.8^a^	64.9^b^	69.2^a^	68.2^a^	66.17^b^	67.46	12.65	1.45
		Feb	64.1^ab^	59.1^c^	67.57^a^	64.8^ab^	62.67^b^	63.65	13.91	3.57
	Mean		68.5^b^	64.7^d^	71.1^a^	67.2^cb^	66.2^c^	67.5	10.67	1.44
	Spring	Mar	66.2^a^	57.6^b^	65.3^a^	61.97^ab^	57.3^b^	61.68	14.12	4.85
First peak Harvest		Apr	80.6^a^	72.0^d^	80.4^a^	78.43^b^	74.8^c^	77.25	7.55	1.82
		May	82.1^a^	75.7^c^	82.6^a^	77.87^b^	78.6^b^	79.37	9.36	2.08
	Mean		76.3^a^	68.4^c^	76.1^a^	72.8^b^	70.2^c^	72.8	9.08	1.87
	Summer	June	84.37^a^	71.0^d^	76.0^c^	81.3^ab^	78.3^bc^	78.21	11.5	3.64
		July	72.3^a^	65.73^cd^	69.47^b^	65.23^d^	67.73^bc^	68.83	10.73	2.34
		Aug	73.57^a^	72.5^a^	73.17^a^	70.4^a^	72.4^a^	71.67	10.29	3.17
	Mean		76.7^a^	69.7 + ^c^	72.86^b^	72.3^b^	72.8^b^	72.9	9.56	2.06

Different letters in the same columns indicate the difference between the two mean statistically significant (P < 0.05) for each treatment combination.

In the winter, the mean temperature increased by 2 °C (18.46 °C–20.13 °C) as compared to the other three seasons (Fig. 2), and the lowest leaf quality scores of 63.65, 67.46, and 71.45% were recorded below the export quality standard in February, January, and December, respectively. In the cases of clones, the lowest, 71.1, 68.5, 67.2, 66.2, and 64.7% mean leaf quality was scored by clones 11/4, BB-35, 11/56, 12/38, and 6/8 in the winter, at a maximum temperature above 26.35 °C and a drop in rainfall to 745 mm/month on average. The clones cease active shoot growth and get into a 3–4 week resting period in the winter.

In spring, the highest 77.25 and 79.37% leaf qualities were scored in April and May, while the lowest 61.68% leaf quality was recorded in March. Because most clones had not fully recovered from the prolonged winter dry in March (Table 1), Regarding clones, 11/4 scored the highest (82.6%) leaf quality in April and May, followed by clones BB-35 (82.1), 12/38 (78.6), 11/56 (78.43), and 6/8 (75.7%). A rise in precipitation has a direct correlation with the quality of the leaves in May. Also, increased water availability increases the production of new leaves on the bushes [51].

In the summer, the highest leaf quality of 78.21% was recorded in June. Clones suddenly get into a short resting phase (Fig. 2), and the percentage of soft-bhanji leaves increases by 5–10% in July, and the leaf quality drops from 78.21% to 68.83% with decreased temperatures and increased rainfall. Clone BB-35 scored a high of 84.37% leaf quality, followed by clones 11/56 (81.3), 12/38 (78.3), 11/4 (76.0), and 6/8 (71.4%) in June. The monthly leaf quality percentage is directly related to the amount of monthly rainfall and the change in temperature. and The monthly leaf quality percentage decreased in the following order: May > June > April > September > November > August > December; hence, the phenological timing of leaf quality is primarily influenced by monthly temperature and rainfall variations over the course of a year. In the case of a season, summer > spring > autumn; thus, all seasons produce two leaves with a bud composition of at least 72%, except winter (Table 1).

### 3.3 Shoot replacement cycle

The average shoot replacement cycle was significantly affected by seasonal variation (P < 0.0059). In the wet season, clones show a short mean shoot replacement cycle of 7.1, 7.6, 8.1, 8.3, 8.8, 9.5, and 10.5 days was recorded in May, June, November, April, October, December, and September, respectively. August and July have 12.1 and 12.5 day long shoot replacements because of the long, rainy, and cool summer days. A short-shoot replacement cycle exists at maximum temperatures above 23.03 °C and below 26.35 °C, but away from these temperatures, clones reduce their plucking rounds to one or two per month. The shoot extension rate of tea buds decreases markedly as the temperature rises above 22 °C [52].

In the dry harvesting season, the maximum temperature reached 28.34 °C and rainfall decreased to 760 mm/month on average. Clones showed a long shoot replacement cycle of 15.7, 13.6, and 13 days in February, March, and January, respectively, and the percentage of leaf quality decreased and hard-bhanji increased by 4–5% in the winter (Table 1). Hence, clones get into a short resting phase and develop bhanji in between February and March (Figure 2, Figure 3), and the rate of shoot growth is directly proportional to the increase in temperature up to the optimum temperature [26].

In the case of clones, 11/4 and BB-35 have produced three leaves and a bud within six-day intervals, whereas clones 11/56, 6/8, and 12/38 showed short (6.92, 7.08, and 9.58) shoot replacement days in May. On the other hand, clones 6/8, 12/38, BB-35, 114, and 11/56 showed long (16.3, 16.3, 15.8, 15.17, and 15.08) shoot replacement days in February. Clones show higher yield and shorter plucking rounds at maximum temperatures above 23.03 °C and below 26.35 °C, but above 28.34 °C the plant shows a negative effect on the total production of green leaves, And the leaf phenotype was affected by higher maximum temperatures, which significantly decreased net photosynthesis and yields in tea plants [53].

### 3.4 Long years climate variables and seasonal yield pattern of Ethiopian tea plantation

In the southwest of Ethiopia, tea plants were harvested throughout the year at a minimum temperature of 10.53–12.54 °C and a maximum temperature of 23.08–26.35 °C. The tea plant is adapted to a wide range of temperatures, 10–30 °C [54]. Even though the yield is influenced by the monthly minimum and maximum temperatures above 26.35 °C and below 10.38 °C and has a negative impact below 12.5 °C [55] and above 26.6 °C [10], the tea plant produced the highest yield at a temperature range of 23–25 °C [56] and 18–25 °C [27].

Regression analysis was used for time series rainfall, temperature, and tea output data over the 22-year period, and the results are summarized in Table 2. A positive linear association between the rainfall, maximum, and mean temperature is present and significant at 95% r = 16.086 mm y^−1^ (P = 0.005), r = 0.127 °C y^−1^ (P = 0.76), and r = 0.0566 °C y^−1^ (P = 0.0007) (Table 2). On the other hand, the minimum temperature was not significant, declining at 95% (r = -0.0147 °C y^−1^). In this case, the annual rainfall increased by 16.09 mm y^−1^, the maximum and mean temperatures were raised by 0.127 °C and 0.0566 °C y^−1^, respectively, and the green leaf was strongly correlated with the maximum temperature (76%), mean temperature (44.6%), and annual rainfall (32.8%), and the yield was well explained by 85.4% of the observed climate variance and increased by 1287.18 tonnes y^−1^ for the last two decades (Table 2).

**Table 2:** Linear relations of yield and climate variable in southwest Ethiopia tea farm.

Variables	Intercept, β0	β1 Chang x/year	r^2^
Rain	1890.57**	16.086**	0.328
Max Temp	24.24***	0.127***	0.76
Min Temp	11.91***	-0.0147 ^ns^	0.226
Mean	18.08***	0.0566***	0.446
Yield/t	11649.4***	1287.18***	0.854
G Yield/ha	6333.147***	370.57***	0.829

**Significance at p = 0.001. *** High Significance at p = 0.0001.

The tea plant exhibits a positive correlation between rainfall and temperature. Hence, its production depends on stable temperatures and rainfall patterns [57]. The tea bush is grouped as a C_3_ plant and its biomass improved via increased photosynthesis and respiration with elevated CO_2_ levels. However rising temperatures may have a significant impact on this increase [25, 26, 58], and temperatures above 32 °C are unfavorable for optimum photosynthesis [59].

Also, in the last two decades of production volume, Wushwush tea farm increased from 2240 tonnes y^−1^ to 3818.8 tonnes y^−1^, Gumaro increased from 1810.3 tonnes y^−1^ to 2546.2 tonnes y^−1^, and Chewaka tea farm increased from 761.9 to 1402.6 tonnes y^−1^, and the national mean production volume increased from 4258.13 to 7767.6 tonnes y^−1^ (2910.8 tonnes ha^−1^) with a low input of 90–150 Nha^−1^. For this improvement, Wushwush, Gumaro, and Chewaka farms contributed around 49, 33, and 18% of total annual made tea production, respectively. Hence, the southwest highlands show a strong positive relationship with current climate change. Also, we predict that tea plants will have a positive net impact on future climate change, even though the recent cold temperatures that occurred between October and January caused a decline in yield. In particular, a temperature below 10.38 °C has a negative impact on leaf quality and tea yield (Fig. 3).

Depending on altitude, the tea industry has three to four harvesting seasons [60]. In southwest Ethiopia, tea is harvested all year. Based on season, the growth area for seasonal harvesting is categorized into two, i.e., the dry and wet harvesting seasons. The dry harvesting season existed between the middle of December and the middle of March. Over the last 20 years, the annual mean temperature (18.7 °C) has increased by 0.50–1.17 °C month^−1^, while rainfall has decreased by 760 mm/month. The dry harvesting season contributed 18–24.3% of the annual yield. This yield variation depends on the amount of temperature and late-season rainfall that occurred in November and December. A later start or an early end to rainfall both reduced the time crops had to complete their growth cycle and caused reduced yields [61]. Winter's prolonged drought reduced yields by 34.97% from the mean. Also, extreme weather events will decrease tea yield by 11%–35% [62]. Drought reduces plantation productivity by 15–45% [63, 64]. The wet season annual mean temperature dropped by 0.25–0.59 °C/month, and the rainfall increased by 285.75 mm/month; the tea plants are very responsive to monthly climate variation and the bush wakes up from 3–4 weeks of short rest periods in the middle of March (Fig. 3). The sum of the wet harvesting season contributed 75.7–82% of the annual yields.

The wet harvesting season is further subdivided into two peak harvesting seasons. The first peak harvesting season began in April, and the highest peak yield of 11.7% was recorded in June. After a while, the tea plant entered a short resting phase, which was associated with long, rainy summer days and a decline in maximum temperatures, and the yield suddenly dropped by 5.1% (6.6%) in July (Fig. 3). Then the soft banjhi increased, but three or four weeks later, the active shoot populations flourished, and the evenness and greenness of the field returned once more. The first harvesting season contributed 40.2%–42.2% of the annual yields.

The second peak harvesting season begins in September and contributes 35.5–40% of the annual yields. The maximum 11.1% peak yield was recorded in November, and then the yield gradually decreased by 7.6, 6.7, and 4.6% in January, February, and March (Fig. 3). In general, the experimental and long-year yield responses of tea plants show that the response potential of a plant is directly governed by a change of season and shows closely similar patterns to the precipitation of a season. Seasonal variation and climate change are essential phenomena for perennial plants to express, respond cyclically, and produce high-quality green leaves on a regular pattern, and highland tea production will exhibit a positive net benefit from the expected climate change in the future.

## 4 Conclusion

The highlands have two harvesting seasons, i.e., a dry and a wet harvesting season. The dry harvesting season contributes 18.3–24.3% of annual yields, depending on the temperature and late-season rainfall that occurred in November and December. Clones have a long shoot replacement cycle of 13–15.7 days, and hard-banjhi leaves increased by 4–5% as rainfall decreased and maximum temperatures rose above 26.35 °C in winter. The wet harvesting season is further subdivided into two peak harvesting seasons. The first harvest season began in the middle of March, had a short plucking round, and gave a high peak yield in April, May, and June. The season accounts for 40.22–42.2% of the annual yield. The second wet harvesting season starts in early September and lasts until the middle of December. It produced a high 11.1% peak yield in November but gradually decreased to 3.3, 4.4, and 6.5% in winter, and the season contributes to 35.5–40% of the annual yield. Phenological timing, which is closely correlated with seasonal variation, has a direct impact on leaf quality and clone yielding potential. In general, over the last two decades, rainfall, maximum, and mean temperatures all increased by 16.09 mm y^−1^, 0.127 °C, and 0.0566 °C y^−1^, respectively, and the tea plant showed a strong correlation with maximum temperature (76%), whereas mean temperature (44.6%) and annual rainfall (32.8%) correlated weakly. Green leaf production is well explained by around 85.4% of the observed climate variance, with an increase of 1287.18 tonnes y^−1^, and the tea plant will exhibit a positive net benefit from expected climate change in the future.

## Author contribution statement

LineNoBookmarkStart:ID:91 = = Name:Line_manuscript_66].

Adugna Debela: Conceived and designed the experiment.

Tesfaye Benti: Performed the experiments; Wrote the paper.

Yetenayet Bekele: Analyzed and interpreted the data.

Sultan Suleman: Contributed reagents, materials, analysis tools or data.

## Funding statement

This work was supported by the 10.13039/501100010705College of Agriculture and Veterinary Medicine, Jimma University and Wushwush tea farm.

## Data availability statement

Data will be made available on request.

## Declaration of interest's statement

The authors declare no conflict of interest.
